# Microsurgery vs. Gamma Knife Radiosurgery for the Treatment of Brainstem Cavernous Malformations: A Systematic Review and Meta-Analysis

**DOI:** 10.3389/fneur.2021.600461

**Published:** 2021-01-26

**Authors:** Xiangyu Gao, Kangyi Yue, Jidong Sun, Yuan Cao, Boyan Zhao, Haofuzi Zhang, Shuhui Dai, Lei Zhang, Peng Luo, Xiaofan Jiang

**Affiliations:** Department of Neurosurgery, Xijing Hospital, Fourth Military Medical University, Xi'an, China

**Keywords:** radiosurgery, neurosurgery, hemorrhage, brainstem, brain cavernous malformations

## Abstract

**Background:** Brainstem cavernous malformations (BSCMs) are a subset of cerebral cavernous malformations with precarious locations and potentially devastating clinical courses. The effects and outcomes of treating BSCMs by microsurgery or gamma knife radiosurgery (GKRS) vary across studies.

**Methods:** We searched the Medline, Web of Science, The Cochrane Library, PubMed, and China Biology Medicine disc databases for original articles published in peer-reviewed journals of cohort studies reporting on 20 or more patients of any age with BSCMs with at least 80% completeness of follow-up.

**Results:** We included 43 cohorts involving 2,492 patients. Both microsurgery (RR = 0.04, 95% CI 0.01–0.16, *P* < 0.01) and GKRS (RR = 0.11, 95% CI 0.08–0.16, *P* < 0.01) demonstrated great efficacy in reducing the rehemorrhage rate after treatment for BSCMs. The incidence rates of composite outcomes were 19.8 (95% CI 16.8–22.8) and 15.7 (95% CI 11.7–19.6) after neurosurgery and radiosurgery, respectively. In addition, we found statistically significant differences in the median numbers of patients between neurosurgical and radiosurgical cohorts in terms of symptomatic intracranial hemorrhage (ICH; neurosurgical cohorts: median 0, range 0–33; radiosurgical cohorts: median 4, range 1–14; *P* < 0.05) and persistent focal neurological deficit (FND; neurosurgical cohorts: median 5, range 0–140; radiosurgical cohorts: median 1, range 0–3; *P* < 0.05).

**Conclusions:** The reported effects of treating BSCMs by microsurgery or GKRS are favorable for reducing recurrent hemorrhage from BSCMs. Patients in the neurosurgery cohort had a lower incidence of symptomatic ICH, while patients in the radiosurgical cohort had a lower incidence of persistent FND.

## Introduction

Cerebral cavernous malformations (CCMs) are low-flow vascular abnormalities of the brain that are composed of clusters of dilated, thin-walled capillaries filled with hemosiderin deposits. These lesions have an incidence in the range of 0.4% to 0.8% in the general population (1). Common manifestations include seizures, headaches, and intracranial hemorrhage. Brainstem cavernous malformations (BSCMs), which are subsets of CCMs, are rare lesions and account for 20% of all CCMs (2). BSCMs are reportedly (3, 4) associated with higher morbidity and mortality rates than other cavernous malformations because of their particular location. Consequently, hemorrhage ictus can lead to acute deterioration of neurological function and can induce severe symptoms. Therefore, BSCMs should be taken seriously and considered for aggressive treatment.

The optimal treatment for BSCMs remains a matter of debate. Microsurgery is the most common technique developed for the management of BSCMs. In the past few decades, dissection techniques and modern imaging modalities such as diffusion tensor imaging (DTI) and electrophysiological monitoring have developed rapidly. They are essential tools in planning the surgical approach for BSCMs and enable a precise surgical procedure (5). Some studies consider microsurgical removal of a cavernous malformation to represent an effective therapy in experienced hands that is generally associated with good clinical outcomes, both neurologically and in terms of quality of life (QoL) (6). However, the risk for surgical morbidity and mortality is high when the lesion is deep or located in particular areas (4, 7–9). Severe complications include cerebrospinal fluid leakage, infection, cranial nerve or motor dysfunction, and vascular hemorrhage. Therefore, reducing the risk of complications remains a substantial challenge.

In recent years, gamma knife radiosurgery (GKRS) has gradually attracted the attention of researchers and clinicians. Some studies consider radiosurgery an effective treatment option for BSCMs (10, 11). Radiosurgery for BSCMs is warranted because of its expected decreased rebleeding rate and lower morbidity.

However, BSCMs treatments have not been compared in a randomized controlled trial. Therefore, we performed a systematic review and meta-analysis of the available data from the published literature to compare microsurgery with radiosurgery in terms of their efficacy and safety for treating BSCMs.

## Methods

The present study was performed according to the Preferred Reporting Items for Systematic Reviews and Meta-Analyses (PRISMA) guidelines (12) and has been registered in PROSPERO (CRD42020206047).

### Search Strategy

**Figure 1:**
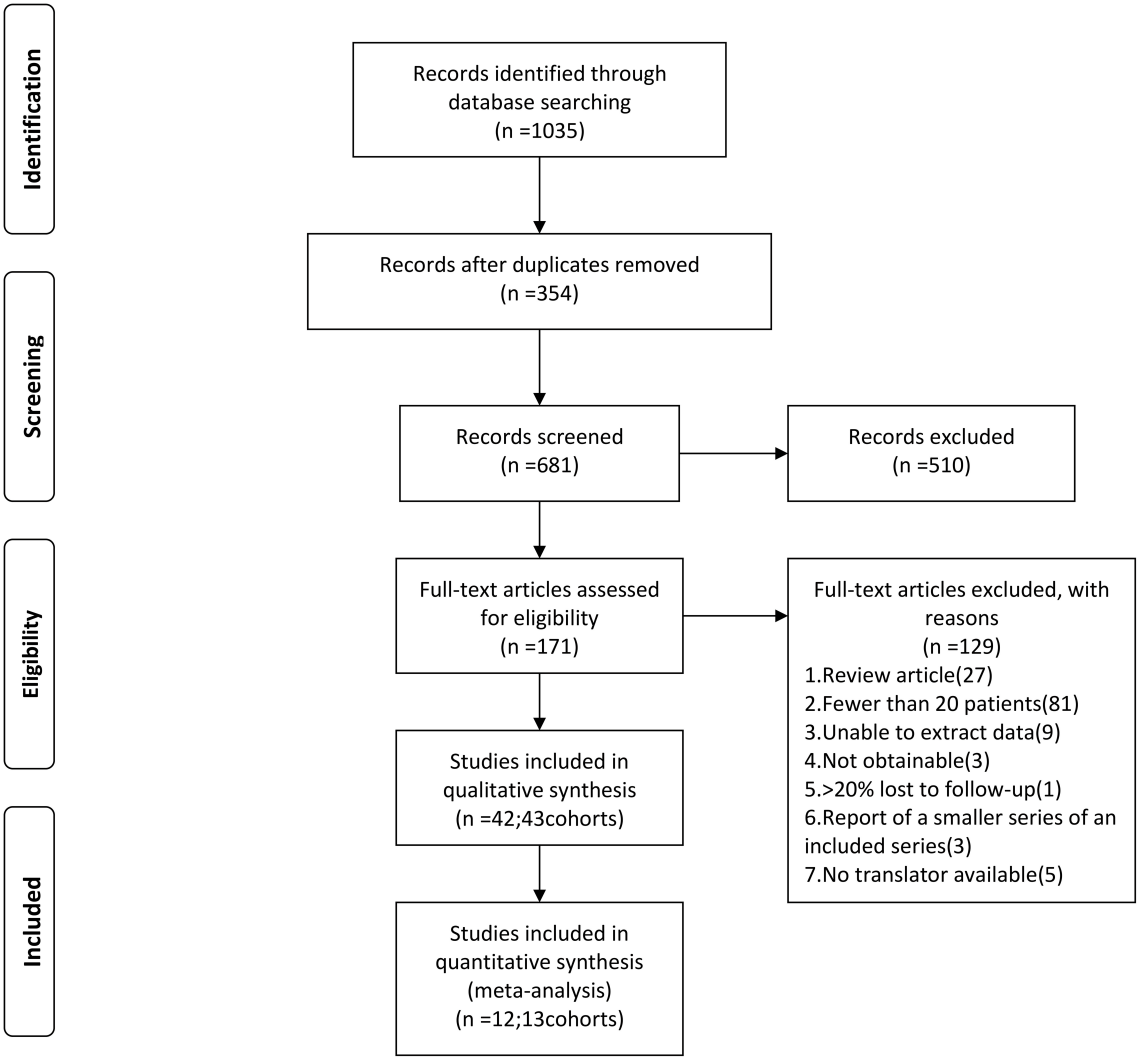
Flow chart of the data search followed by PRISMA guidelines. PRISMA, Preferred Reporting Items for Systematic Reviews and Meta-Analyses.

We searched five databases: Medline, Web of Science, The Cochrane Library, PubMed, and China Biology Medicine disc (January 1990–April 2019). The following keywords were used: “brainstem,” “brain cavernous hemangioma,” “cerebral cavernous malformation,” “cerebral cavernous hemangioma,” and “hemangioma, cavernous, central nervous system.” We retrieved the original articles of cohort studies published in peer-reviewed journals. We included eligible studies published in Chinese and English, while studies in other languages were excluded because we did not have translators (Figure 1).

### Assessment of Eligibility

Two independent reviewers selected eligible studies based on the Patient, Intervention, Comparison, Outcome, and Study design (PICOS) guidelines (13): (1) Participants: patients' BSCMs had to be confirmed by MRI or pathological examination; (2) Interventions: microsurgery or radiosurgery; (3) Comparison: not applicable; (4) Outcome: death, symptomatic intracranial hemorrhage (ICH) and persistent focal neurological deficit (FND); (5) Study designs: retrospective cohort study; the sample sizes of the studies had to be >20; studies must have described the follow-up time, and the follow-up rate had to be >80%. If the institution or author published multiple studies using the same cohort, only the report with the largest sample size was included for analysis. Case reports, reviews, meta-analyses, letters and conference articles were excluded.

### Risk of Bias Assessment

The Newcastle-Ottawa Scale (NOS) was used to assess the quality of the included studies. The NOS score is used to assess three major components: selection, comparability, and exposure. Studies are defined as high quality when scoring ≥5. Two reviewers independently evaluated the quality of the studies and resolved disagreements by discussion.

### Data Extraction

A total of 1,035 articles were initially identified. Two reviewers (Xiangyu Gao and Peng Luo) independently screened the 1,035 articles, eventually excluding 993 and leaving 42. All the discrepancies were ultimately agreed upon through discussion. We collected data on patient demographics, duration of follow-up, and type of BSCMs treatment (6, 9–11, 14–51) (Table 1). We quantified the occurrence of composite outcomes including death, symptomatic ICH and persistent FND caused by BSCMs or their treatment during follow-up.

**Table 1:** Basic patient characteristics of each included cohort.

**Reference**	**Number of treated patients**	**Mean duration of follow-up (months)**	**Mean age (years) (range)**	**Number of female patients (%)**	**Number of patients with multiple BSCMs (%)**	**Mean size (mm)(range)**
**Neurosurgery (*****n*** **=** **35)**						
Porter et al. (14)	100	35 (–)	37 (3–64)	62 (62)	24 (100)	15 (1–45)
Samii et al. (15)	36	18 (3–60)	35.8 (7–64)	18 (50)	–	10.4 (3.5–16.3)
Ferroli et al. (16)	52	51.6 (18–126)	38.5 (3–70)	26 (50)	7 (13)	–
Bruneau et al. (17)	22	44.9 (–)	39.8 (10–66.4)	7 (32)	4 (18)	–
Hauck et al. (18)	44	–	37.5 (10–77)	30 (68)	6 (14)	–
Li et al. (19)	37	21.5 (6–36)	36.5 (18–58)	25 (68)	–	–
Abla et al. (20)	40	31.9 (–)	12.3 (0.8–18.9)	21 (53)	–	23 (–)
Huang et al. (21)	30	48.5 (–)	40.4 (7–70)	15 (50)	1 (3)	13.5 (–)
Mai et al. (22)	23	38 (–)	32.6 (15–67)	13 (57)	–	11 (4–21)
Ohue et al. (23)	36	–	42 (–)	24 (67)	–	20 (7–30)
Chen et al. (24)	55	49 (–)	37.2 (11–63)	26 (47)	–	–
Dukatz et al. (6)	71	17 (6–100)	36 (13–69)	33 (46)	3 (4)	–
Ma et al. (25)	26	– (8–42)	35.6 (15–67)	11 (42)	–	–
Menon et al. (26)	23	42 (–)	25.4 (11–58)	–	2 (9)	–
Zhang et al. (27)	41	38 (6–72)	35.5 (8–62)	18 (44)	–	–
Bradac et al. (28)	37	39 (–)	34.7 (–)	16 (43)	12 (32)	–
Chen et al. (29)	46	9.7 (3–58)	38.6 (15–69)	27 (59)	–	–
Li et al. (40)	242	89.4 (4.4–170.4)	33 (3–64)	104 (43)	28 (12)	19 (–)
Mai et al. (49)	22	26.6 (4–68)	43 (8–69)	15 (68)	6 (27)	5.4 (–)
Schwartz et al. (50)	35	44 (8–115)	39.3 (7–70)	20 (57)	11 (31)	16 (6–27)
Chen et al. (51)	38	9.7 (–)	36.2 (15–61)	23 (61)	–	–
Frischer et al. (30)	29	115.2 (24–265.2)	37.4 (1.6–63.7)	13 (45)	6 (21)	7.8 (2.9–16.1)
Li et al. (31)	52	94.8 (3.6–213.6)	14.2 (–)	15 (29)	7 (13)	21 (11–34)
Garcia et al. (32)	104	18.6 (–)	42.1 (–)	58 (56)	–	19.5 (–)
Gu (33)	50	9.7 (–)	33 (24–72)	21 (42)	–	–
Liu et al. (34)	22	24 (–)	41.9 (18–69)	14 (64)	–	–
Wang et al. (35)	23	42 (3–96)	41 (15–62)	10 (43)	–	–
Farhoud and Aboul-Enein et al. (36)	24	45 (–)	34 (12–58)	14 (58)	3 (13)	–
Zhang et al. (37)	120	50.7 (18–90)	40.3 (4–69)	64 (53)	–	20.4 (5–36)
Nathal et al. (38)	50	33 (–)	35.9 (–)	29 (58)	2 (4)	18 (6.6–31.4)
Ren et al. (39)	34	67.2 (–)	38.6 (18–60)	18 (53)	6 (18)	17 (–)
Zaidi et al. (40)	397	35.5 (–)	42.2 (–)	237 (60)	–	17.7 (–)
Gui et al. (41)	67	51.7 (40–66)	40 (14–68)	35 (52)	–	–
Xie et al. (42)	69	35.3 (–)	32.6 (–)	30 (43)	–	18.2 (–)
Zhang et al. (43)	25	8 (3–15)	40 (8–61)	8 (32)	–	23 (–)
**Radiosurgery(*****n*** **=** **8)**						
Zhu et al. (44)	34	– (5–72)	42 (14–64)	16 (47)	–	12.8 (8–18)
Monaco et al. (9)	68	62.4 (7.2–148.8)	41.2 (5–79)	34 (50)	–	–
Park and Hwang (45)	21	38.9 (18–82)	41.1 (24–69)	9 (43)	3 (14)	–
Frischer et al. (30)	38	62.4 (25.2–177.6)	43.7 (19.3–76.8)	19 (50)	3 (8)	4.2 (2.9–8.4)
Kim et al. (11)	39	44.4 (3.6–190.8)	41.5 (18–64)	23 (59)	3 (8)	–
Liu et al. (46)	43	36 (12–120)	41.7 (22–66)	23 (53)	–	–
Park et al. (47)	45	111.7 (61.2–232.8)	36.6 (3–67)	31 (69)	–	–
Kefeli et al. (10)	82	50 (13–113)	41.5 (–)	35 (43)	6 (7)	5.3 (1–36)

*BSCMs, brainstem cavernous malformations. We used median, if mean was not available*.

### Statistical Analysis

We quantified the number of patients with outcome events during follow-up and calculated the outcome event incidence and 95% CIs per patient. We prespecified the following characteristics of the included cohorts as the baseline covariates of interest: mean age of the patients at the time of surgery, the percentage of female patients, cohort midyear (defined as the middle of the year in which the treatment occurred), proportion of patients with multiple BSCMs, mean size of the BSCMs, BSCMs location and median number of patients with outcome events. We used the Mann-Whitney U test to evaluate the differences in the proportions of these characteristics between the neurosurgical and radiosurgical cohorts, with a *P* < 0.05 indicating a significant difference. To standardize the evaluation of the research results, we counted the occurrence of hemorrhage during the total person-years of follow-up indicated or by multiplying the median or mean follow-up time by the total number of patients treated. The pretreatment and posttreatment hemorrhagic rates were calculated separately, and the risk ratio (RR) was computed. An RR<1 indicates that the treatment reduced the risk of rebleeding. Meta-analysis software (version 5.3, Review Manager) was used to calculate the overall RR. Differences with *P* < 0.05 were considered statistically significant. Statistical heterogeneity among the included studies was evaluated by I^2^. If we observed I^2^ > 50%, we used a random-effects model to analyze the assumption. Otherwise, we used a fixed-effects model. Sensitivity analysis was performed to investigate the impact of an individual study on the overall risk assessment by omitting one study per round. Publication bias was evaluated by funnel plot regression.

## Results

### Systematic Literature Review

A total of 2,492 patients were enrolled in 42 eligible studies, including 43 cohorts (Table 2). Eight cohorts involving 370 patients were included in reports on GKRS, and 2,122 patients in 35 cohorts were included in reports on neurosurgery. Thirty-nine cohorts (91%) described the mean or median duration of follow-up. The patients' most common symptoms were cranial nerve dysfunction, sensory disturbances, motor palsy, hemiparesis, and headache. We found statistically significant differences in the proportions of patients between the neurosurgical and radiosurgical cohorts in terms of the mean age of the patients at the time of surgery, the mean size of the BSCMs and the BSCM location. GKRS was more suitable for older patients (neurosurgical cohorts: median 37.2, range 12.3–43; radiosurgical cohorts: median 41.6, range 36.6–43.7; *P* < 0.01), while neurosurgery was more suitable for patients with larger lesions (neurosurgical cohorts: median 17.8, range 5.4–23; radiosurgical cohorts: median 5.3, range 4.2–12.8; *P* < 0.05). When considering the locations of the lesions in patients, neurosurgery was more suitable for the midbrain (neurosurgical cohorts: median 22, range 0–93; radiosurgical cohorts: median 14, range 8–24; *P* < 0.05) and pons (neurosurgical cohorts: median 62, range 7–83; radiosurgical cohorts: median 57, range 44–61; *P* < 0.05), while GKRS was more suitable for the medulla (neurosurgical cohorts: median 14, range 0–79; radiosurgical cohorts: median 26, range 0–35; *P* < 0.05).

**Table 2:** Characteristics of the included cohorts.

	**Overall (*****n*** **=** **43)**			**Neurosurgery (*****n*** **=** **35)**			**Radiosurgery (*****n*** **=** **8)**		
**Study characteristics**	**Cohorts (%)^†^**	**Patients**	**Median (range)**	**Cohorts (%)**	**Patients**	**Median (range)**	**Cohorts (%)**	**Patients**	**Median (range)**
Patients treated	43 (100)	2,492	39 (21–397)	35 (100)	2,122	38 (22–397)	8 (100)	370	41 (21–82)
Duration of follow-up, y	39 (91)	2,352	3.3 (0.7–9.6)	32 (91)	2,016	3.2 (0.7–9.6)	7 (88)	336	4.2 (3–9.3)
Mid-year, y	43 (100)	2,492	2004 (1990–2014)	35 (100)	2,122	2005 (1990–2014)	8 (100)	370	2004 (1996–2011)
Age, y	43 (100)	2,492	38.5 (12.3–43.7)	35 (100)	2,122	37.2 (12.3–43)^**^	8 (100)	370	41.6 (36.6–43.7)^**^
Female, %	42 (98)	2,469	51 (29–69)	34 (97)	2,099	52 (29–68)	8 (100)	370	50 (43–69)
Multiple BSCMs, %	20 (47)	1,047	13 (3–32)	16 (46)	867	13 (3–32)	4 (50)	180	8 (7–14)
Size, mm	21 (49)	1,598	17 (4.2–23)	18 (51)	1,444	17.8 (5.4–23)^*^	3 (38)	154	5.3 (4.2–12.8)^*^
**BSCM location**									
Midbrain, %	38 (88)	2,327	22 (0–93)	31 (89)	2,025	22 (0–93)^*^	7 (88)	302	14 (8–24)^*^
Pons, %	38 (88)	2,327	61 (7–83)	31 (89)	2,025	62 (7–83)^*^	7 (88)	302	57 (44–61)^*^
Medulla, %	38 (88)	2,327	16 (0–79)	31 (89)	2,025	14 (0–79)^*^	7 (88)	302	26 (0–35)^*^

* P < 0.05 and

** *P < 0.01, showing a significant difference in the median ratio between the group describing neurosurgery and the group describing radiosurgery. †The percentage is the number of cohorts reporting a particular study characteristic divided by the total number of cohorts. BSCMs, brainstem cavernous malformations*.

### Hemorrhage Rate

**Figure 2:**
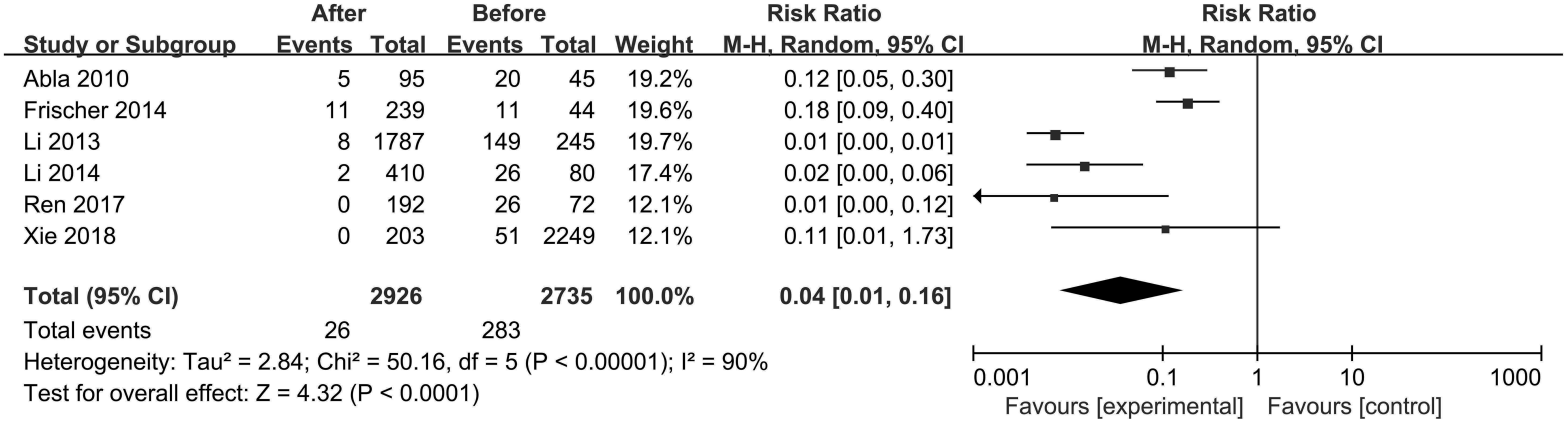
Forest plot of neurosurgery studies comparing the RRs.

**Figure 3:**
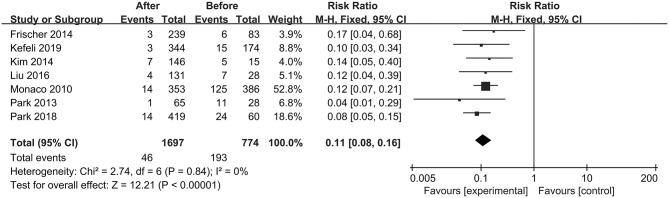
Forest plot of radiosurgery studies comparing the RRs.

The first preoperative hemorrhage was defined as the clinical manifestation of BSCMs and was excluded. Therefore, we counted the annual preoperative rehemorrhage rate and used it to calculate RR (Table 3). Thirteen cohort studies described preoperative and postoperative rehemorrhage rates. Of the six cohort studies on neurosurgery, all demonstrated the efficacy of surgery in the treatment of BSCMs (RR < 1). When all of the neurosurgical cohort studies were analyzed, the overall RR was 0.04 (95% CI 0.01–0.16, *P* < 0.01), which suggested that neurosurgery can significantly reduce the risk of rebleeding. The seven cohort studies on GKRS also demonstrated great efficacy in the treatment of BSCMs (RR < 1). The overall RR of GKRS was 0.11 (95% CI 0.08–0.16, *P* < 0.01). When all six neurosurgery studies were analyzed together, there was some heterogeneity (*P* < 0.00001; I^2^ = 90%). Thus, the assumptions were analyzed using a random-effects model due to I^2^ > 50%. However, all GKRS studies were analyzed using a fixed-effects model (*P* < 0.00001; I^2^ = 0) (Figures 2, 3).

**Table 3:** Reported risk of hemorrhage in 13 included cohorts.

**Reference**	**Number of treated patients**	**Mean duration of follow-up (months)**	**Hemorrhage rate (%)**	
			**Before treatment**	**After treatment**
**Neurosurgery (*****n*** **=** **6)**				
Abla et al. (20)	40	31.9 (–)	44.0	5.2
Li et al. (48)	242	89.4 (4.4–170.4)	60.8	0.4
Frischer et al. (30)	29	115.2 (24–265.2)	25.0	4.6
Li et al. (31)	52	94.8 (3.6–213.6)	32.5	0.4
Ren et al. (39)	34	67.2 (–)	36.1	0
Xie et al. (42)	69	35.3 (–)	2.3	0
**Radiosurgery (*****n*** **=** **7)**				
Monaco et al. (9)	68	62.4 (7.2–148.8)	32.4	4.0
Park and Hwang (45)	21	38.9 (12–82)	39.3	1.5
Frischer et al. (30)	38	62.4 (25.2–177.6)	7.2	1.3
Kim et al. (11)	39	44.4 (3.6–190.8)	33.3	4.8
Liu et al. (46)	43	36 (12–120)	25.0	3.1
Park et al. (47)	45	111.7 (61.2–232.8)	40.0	3.3
Kefeli et al. (10)	82	50 (13–113)	8.6	0.9

### Incidence of Composite Outcomes

The numbers of cohorts reporting on the different outcome events are displayed in Table 4. Composite outcomes include death, symptomatic ICH and persistent FND. The criteria for postoperative symptomatic ICH were new hemorrhage in the BSCMs or adjacent brainstem parenchyma on CT or MRI with any new or worsening neurological deficits. Twenty-one (49%) cohorts involving 1,008 patients described postoperative composite outcomes (14 neurosurgery cohorts involving 683 patients and 7 radiosurgery cohorts involving 325 patients). There was no statistically significant difference in the numbers of patients between neurosurgical (median 4, range 0–78) and radiosurgical (median 5, range 1–22) cohorts in terms of composite outcomes. There was also no statistically significant difference in the number of patients between the neurosurgical (median 0, range 0–4) and radiosurgical (median 0, range 0–2) cohorts in terms of deaths attributable to BSCM or treatment.

**Table 4:** Incidence of composite outcome.

		**Cohorts (%)**	**Patients**	**Number of patients with outcome events**	**Median number of patients per cohort (range)**	**Outcome event incidence (95%CI) per patient (%)**
All cohorts	Follow-up	43 (100)	2,492	–	–	–
	Composite outcome^†^	21 (49)	1,008	186	4 (0–78)	18.5 (16.1–20.8)
	Deaths attributable to BSCM or treatment	41 (95)	2,415	25	0 (0–4)	1.0 (0.6–1.4)
	Deaths not attributable to BSCM or treatment	38 (88)	1,858	17	0 (0–5)	0.9 (0.5–1.3)
	Symptomatic ICH	29 (67)	1,711	97	1 (0–33)	5.7 (4.6–6.8)
	Persistent FND	31 (72)	1,966	341	3 (0–140)	17.3 (15.7–19.0)
Neurosurgery cohorts	Follow-up	35 (100)	2,122	–	–	–
	Composite outcome	14 (40)	683	135	4 (0–78)	19.8 (16.8–22.8)
	Deaths attributable to BSCM or treatment	33 (94)	2,045	23	0 (0–4)	1.1 (0.7–1.6)
	Deaths not attributable to BSCM or treatment	30 (86)	1,488	12	0 (0–5)	0.8 (0.4–1.3)
	Symptomatic ICH	21 (60)	1,341	59	0 (0–33)^*^	4.4 (3.3–5.5)
	Persistent FND	24 (69)	1,641	335	5 (0–140)^*^	20.4 (18.5–22.4)
Radiosurgery cohorts	Follow-up	8 (100)	370	–	–	–
	Composite outcome	7 (88)	325	51	5 (1–22)	15.7 (11.7–19.6)
	Deaths attributable to BSCM or treatment	8 (100)	370	2	0 (0–2)	0.5 (0–1.3)
	Deaths not attributable to BSCM or treatment	8 (100)	370	5	0 (0–5)	1.4 (0.2–2.5)
	Symptomatic ICH	8 (100)	370	38	4 (1–14)^*^	10.3 (7.2–13.4)
	Persistent FND	7 (88)	325	6	1 (0–3)^*^	1.8 (0.4–3.3)

* *P < 0.05, showing a significant difference in the median ratio between the group describing neurosurgery and the group describing gamma knife radiosurgery*.

† *Composite outcome consisted of death, symptomatic ICH, persistent FND. BSCM, brainstem cavernous malformation; ICH, intracranial hemorrhage; FND, focal neurological deficit; CI, confidence interval*.

In addition, we found statistically significant differences in the numbers of patients between neurosurgical and radiosurgical cohorts in terms of symptomatic ICH and persistent FND. Twenty-one of the 35 (60%) neurosurgical cohorts described postoperative symptomatic ICH, and the remaining neurosurgical cohorts did not describe the hemorrhage of patients after neurosurgery. All 8 (100%) radiosurgical cohorts described postoperative symptomatic ICH. There was a larger number of patients experiencing symptomatic ICH in radiosurgical cohorts (neurosurgical cohorts: median 0, range 0–33; radiosurgical cohorts: median 4, range 1–14; *P* < 0.05). Twenty-four of the 35 (69%) neurosurgical cohorts and 7 of 8 (88%) radiosurgical cohorts described postoperative persistent FND. There was a larger number of patients experiencing persistent FND in the neurosurgical cohorts (neurosurgical cohorts: median 5, range 0–140; radiosurgical cohorts: median 1, range 0–3; *P* < 0.05).

### Sensitivity Analysis

Sensitivity analysis was performed to investigate the impact of a single study on the overall risk assessment by omitting one study in each round. The comparison results were not significantly changed, indicating that our results were statistically reliable.

### Publication Bias

**Figure 4:**
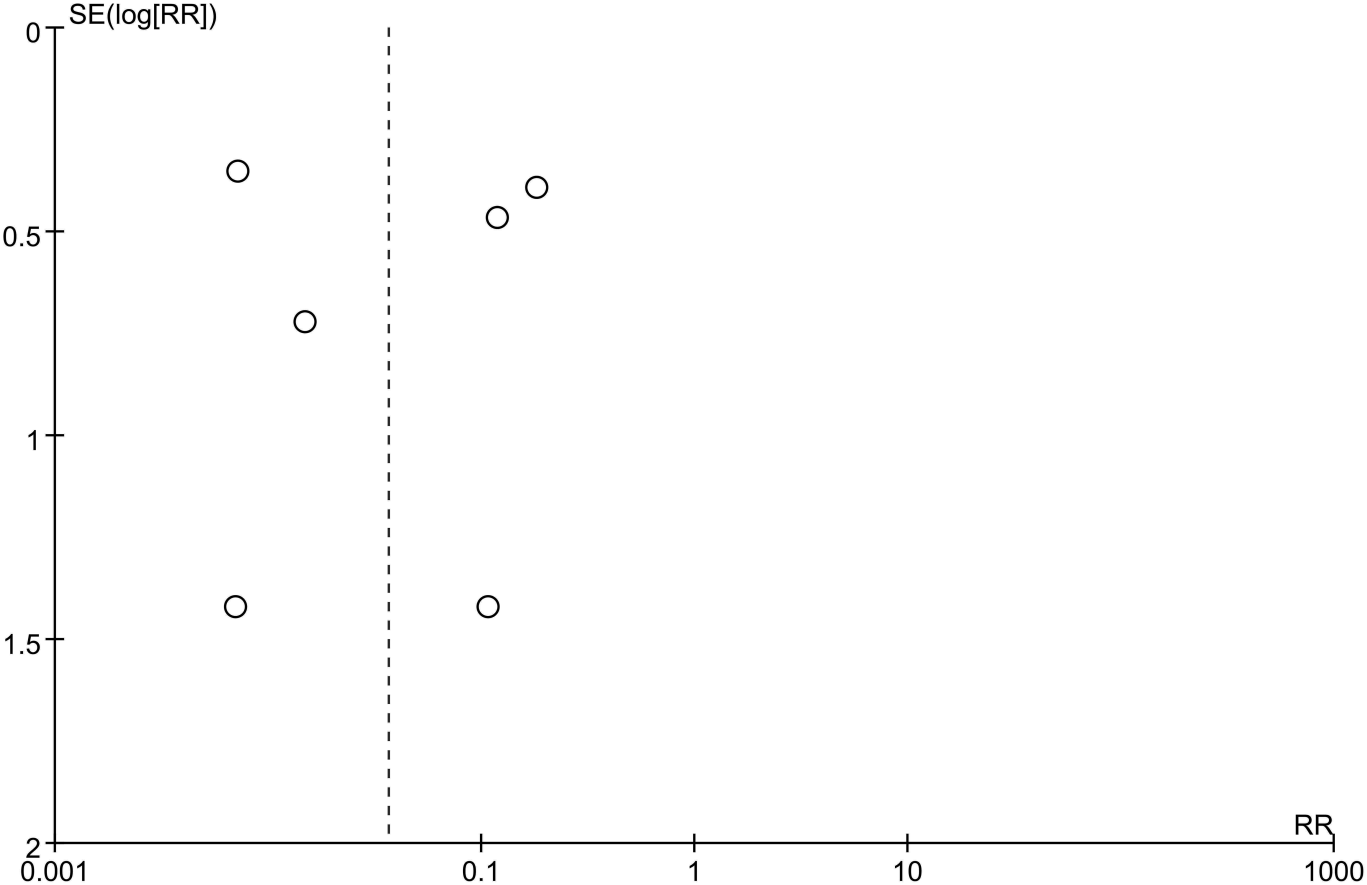
Funnel plot of neurosurgery studies.

**Figure 5:**
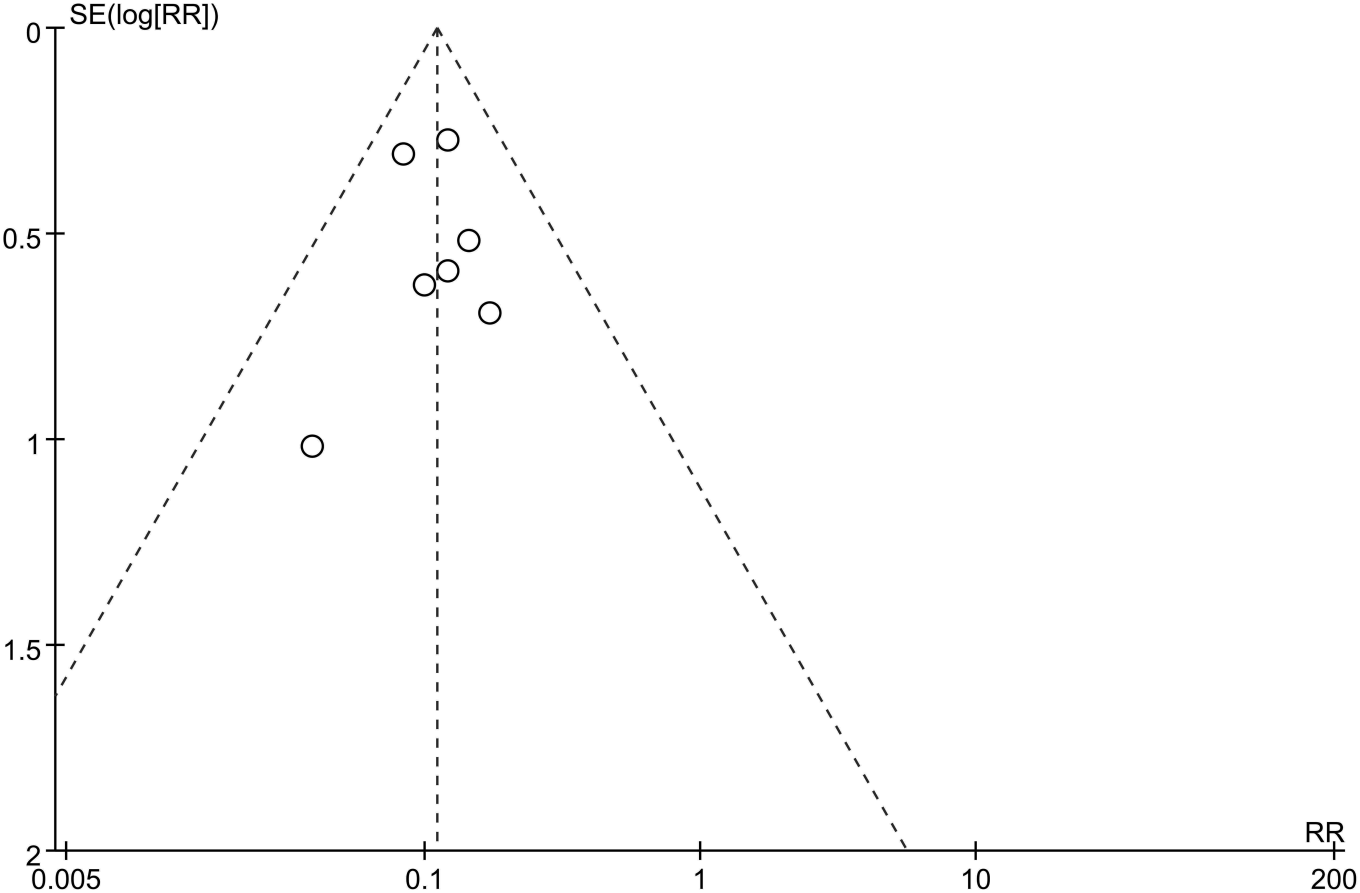
Funnel plot of radiosurgery studies.

Funnel plots were used to assess potential publication bias. No significant funnel asymmetry was observed in any comparison, suggesting that our findings were unlikely to be influenced by significant publication bias (Figures 4, 5).

## Discussion

Brainstem cavernous malformations are a major cause of brainstem hemorrhage, especially if they are not accompanied by coma. The rate of first hemorrhage in BSCMs is only 0.6~1.1%, but the rate of rehemorrhage in BSCMs can be as high as 30~60%. As rehemorrhage occurs, the time interval between successive hemorrhages becomes increasingly shorter. With each rehemorrhage, the patient's symptoms become progressively worse, and patients are increasingly less likely to recover from neurological symptoms (52). Therefore, the natural history of BSCMs is an indication that timely and correct management is critical to prevent hemorrhage.

In 1928, Dandy performed the first neurosurgical procedure to treat BSCMs. At present, it is believed that the neurosurgical indications for BSCMs include a history of bleeding, obvious clinical symptoms, and compliance with the neurosurgical approach (21, 53). Most of the current literature reports that neurosurgery can effectively treat BSCMs and improve patient prognosis (39, 48, 54). Additionally, neurosurgery should be performed in the subacute phase of the disease (from weeks to 1 month after the onset of the disease), as this time period makes it easier to differentiate hematoma from CM lesions during neurosurgery (40). The choice of neurosurgical approach mainly includes the suboccipital fourth ventricle bottom approach, the posterior sigmoid sinus approach, the anterior sigmoid sinus approach, the inferior temporal approach, the Kawase approach, the far lateral approach and the superior cerebellar approach (55, 56). With the application of MRI, electrophysiological monitoring and neuronavigation, the effect and safety of neurosurgery are getting better, and neurosurgery has become the first choice for treating BSCMs (37, 57–60).

In addition to neurosurgery, stereotactic radiosurgery may be considered for lesions that are deep and inaccessible for neurosurgery (46, 61). There has been a considerable amount of research on the effectiveness of this modality. Kefeli et al. showed that GKRS could safely treat BSCMs and effectively decrease the risk of rehemorrhage (10). However, stereotactic radiosurgery is still controversial. The rate of rebleeding in cavernous malformations without stereotactic radiosurgery also decreased significantly 2 years after the initial bleeding, similar to the decrease in rebleeding caused by stereotactic radiosurgery. In addition, the pathological examination of some lesions treated with neurosurgery for rebleeding after radiosurgery did not reveal the pathological basis for the reduction in the rebleeding rate caused by the occlusion of the vascular lumen caused by radiosurgery. Moreover, radiosurgery can cause edema in the brain stem and risks the development of cavernous malformations.

To compare the efficacy and safety of neurosurgery and radiosurgery for BSCMs, we conducted a systematic review and meta-analysis of available data from published literature. The results of our systematic review showed that neurosurgery is more likely to be used in patients with larger lesions and BSCMs lesions located in the midbrain and pons, while radiosurgery is more likely to be used in older patients and patients with BSCM lesions located in the medulla. In addition, both neurosurgery and radiosurgery may bring some risk of postoperative complications. We found that there was no significant difference in composite outcome between the two treatments, but the number of patients with persistent FND was significantly higher in the neurosurgery group than in the radiosurgery group, and the number of patients with symptomatic ICH was significantly higher in the radiosurgery group than in the neurosurgery group. Therefore, further random controlled trials are needed to study the specific advantages and disadvantages of the two treatments.

The change of hemorrhage rate before and after operation is an important index to evaluate the treatment effect of BSCMs. Compared with the postoperative hemorrhage rate, it is very challenging to estimate the accurate preoperative hemorrhage rate of BSCMs. There are three methods to calculate the preoperative hemorrhage rate. First, the preoperative hemorrhage rate is calculated based on the assumption that BSCMs exist from birth. Second, the preoperative hemorrhage rate is calculated based on the assumption that the beginning of the observation period is retrospectively defined as the time of the initial symptoms. Third, the preoperative rehemorrhage rate is calculated based on the assumption that the first hemorrhage is defined as the clinical manifestation of BSCMs and is excluded. Since the first two methods of calculating preoperative hemorrhage rates could not be used for meta-analysis, as the data were not provided in the majority of the included studies, the third method was used to calculate the preoperative rehemorrhage rate in the present meta-analysis. The results of our meta-analysis showed that both microsurgery (RR = 0.04, 95% CI 0.01–0.16, *P* < 0.01) and GKRS (RR = 0.11, 95% CI 0.08–0.16, *P* < 0.01) demonstrated great efficacy in reducing the rehemorrhage rate after treatment for BSCMs. However, this calculation method is still worth exploring. Some lesions may have bled, but this has not been found in other patients with less bleeding who were not referred to a referral center. Regarding rehemorrhage after the initial hemorrhage, the calculated annual rehemorrhage rate before the referral between the initial hemorrhage and subsequent hemorrhage may be overestimated, considering the referral of patients with aggressive disease (20). Hence, calculating the first-ever hemorrhage rate and the rehemorrhage rate separately might be the better method for evaluating the hemorrhage rate. Unfortunately, the majority of cohort studies lacked detailed data to calculate the first-ever hemorrhage rates, which restricted the application of this method in meta-analysis.

The NOS was used to assess the quality of the included studies, and each study had a moderate level of quality, with an average score of 5. Our systematic review and meta-analysis has three limitations. First, all the included studies were retrospective studies due to the lack of randomized controlled trials. Therefore, we could only compare the two treatments indirectly instead of directly. Additionally, randomized controlled trials are urgently needed. Second, because of the different approaches chosen by surgeons, neurosurgery is inconsistent in all included studies, which may lead to some discrepancies in the data. Last, the characteristics of surgery and radiosurgery are different. The outcome of the neurosurgery is greatly affected by the experience of surgeons while the outcome of radiosurgery is not affected by operator's skill as long as standard methods are used. These different characteristics may lead to data bias.

## Conclusion

In conclusion, the treatment of BSCMs is generally more likely to favor neurosurgery. However, as an alternative treatment, stereotactic radiosurgery is of great value in the treatment of BSCMs. Therefore, relevant clinical randomized controlled trials should be carried out, as they would be of great significance for exploring the value of different surgical methods in the treatment of BSCMs.

## Data Availability Statement

The original contributions presented in the study are included in the article/supplementary materials, further inquiries can be directed to the corresponding author/s.

## Author Contributions

XG, KY, and JS contributed to the conception and design of the study. PL and XJ organized the database. YC performed the statistical analysis. XG wrote the first draft of the manuscript. BZ, HZ, SD, LZ, and PL wrote sections of the manuscript. All authors contributed to the manuscript revision and read and approved the submitted version.

## Conflict of Interest

The authors declare that the research was conducted in the absence of any commercial or financial relationships that could be construed as a potential conflict of interest.
